# The Effect of Hindwing Trajectories on Wake–Wing Interactions in the Configuration of Two Flapping Wings in Tandem

**DOI:** 10.3390/biomimetics9070406

**Published:** 2024-07-04

**Authors:** Xu He, Chao Wang, Pan Jia, Zheng Zhong

**Affiliations:** 1 School of Science, Harbin Institute of Technology (Shenzhen), Shenzhen 518055, China; hexu19950207@163.com (X.H.); zhongzheng@hit.edu.cn (Z.Z.); 2 School of Mechanical Engineering, Dongguan University of Technology, Dongguan 523808, China

**Keywords:** tandem wings, forward flight, trajectory, wake–wing interactions

## Abstract

The present investigations on tandem wing configurations primarily revolve around the effects of the spacing *L* and the phase difference φ between the forewing and the hindwing on aerodynamic performance. However, in nature, organisms employing biplane flight, such as dragonflies, demonstrate the ability to achieve superior aerodynamic performance by flexibly adjusting their flapping trajectories. Therefore, this study focuses on the effects of φ, as well as the trajectory of the hindwing, on aerodynamic performance. By summarizing four patterns of wake–wing interaction processes, it is indicated that $\varphi =-90^{\circ }$ and $0^{\circ }$ enhances the thrust of the hindwing, while $\varphi =90^{\circ }$ and $180^{\circ }$ result in reductions. Furthermore, the wake–wing interactions and shedding modes are summarized corresponding to three kinds of trajectories, including elliptical trajectories, figure-eight trajectories, and double figure-eight trajectories. The results show that the aerodynamic performance of the elliptical trajectory is similar to that of the straight trajectory, while the figure-eight trajectory with positive surging motion significantly enhances the aerodynamic performance of the hindwing. Conversely, the double-figure-eight trajectory degrades the aerodynamic performance of the hindwing.

## 1. Introduction

In nature, birds and insects can achieve exceptional aerodynamic performance through wing flapping [1,2]. They can dynamically adjust wing trajectories, enabling various flight modes such as hovering, gliding, and agile maneuvers [3,4,5,6,7,8]. Therefore, the unsteady aerodynamics associated with flapping wings have garnered significant attention from researchers of Micro Air Vehicles (MAVs) [9,10,11,12,13,14]. In nature, most birds and insects generate reverse von Karman vortex street through flapping wings, obtaining thrust from this process [15]. However, not all flapping motions result in a reverse von Karman vortex street. Guyon et al. [15] pointed out that the generation of reverse von Karman vortices depends largely on the Strouhal number (St). In the range of 0.2≤St≤0.4, significant reverse von Karman vortex streets are observed during flapping, and this range corresponds to the typical motions of many birds, insects, and fish [15,16]. At St≈0.15, there is a transition from s reverse von Karman vortex street to regular von Karman vortex street. When St≥0.4, the vortex street will lose its periodicity [15]. Similar results have been observed in the experimental work conducted by Lua et al. [17]. As the Strouhal number (St) varied and the flapping frequency decreased, the wake in the flow field exhibited patterns such as a regular von Karman vortex street, a reverse von Karman vortex street, a neutral wake, dissipated wake, and deflected wake. Another experiment conducted by Anderson et al. [18] suggests that the reverse von Karman vortex street not only enhances thrust but also increases thrust efficiency.

Although most flying organisms usually generate thrust by flapping a pair of wings, some species can achieve outstanding aerodynamic performance through tandem wings, as observed in dragonflies and damselflies [19,20]. This perspective leads researchers to explore the aerodynamic characteristics of tandem wings. For tandem wing configurations, a critical parameter is the phase angle difference between the forewing and hindwing kinematics (φ) [21,22]. Some observations indicate that dragonflies generally adopt a phase difference φ of $55^{\circ }$–$100^{\circ }$ during straight forward flight [23,24], while they use a phase difference of $180^{\circ }$ during hovering [25,26]. Additionally, observations by Thomas et al. [27] suggest that dragonflies also use a phase difference of $180^{\circ }$ during forward flight. While recent research [28,29,30,31,32,33] suggests that, with an appropriate distance between the fore and hind wings, better aerodynamic performance can be achieved with a phase difference (φ) near $0^{\circ }$. For example, Lua et al. [29], combining experiments and simulations, found that with a fixed spacing of L=2c (*L* is the center-to-center wing spacing, *c* is the wing chord length), when $\varphi \in (-90^{\circ },90^{\circ })$, the performance of the 2D tandem wing configuration surpasses the sum of the two individual wing configurations. Moreover, the maximum thrust of the hindwing occurs at $\varphi =0^{\circ }$. Two types of wing–wake interactions are identified based on whether the hindwing penetrates the shear layer of the forewing wake. Following that, simulations by Muscutt et al. [31] also indicated that altering φ and *L* could transition the hindwing from producing no thrust to generating twice the thrust of a single wing. However, when $\varphi =180^{\circ }$, the average thrust produced by tandem wings is lower than that of a single wing. Experiments by Nagai et al. [34] indicate that although tandem wings exhibit maximum aerodynamic efficiency when $\varphi =0^{\circ }$, it is disadvantageous for generating vertical and horizontal aerodynamic forces during hovering and slow forward flight when $\varphi \neq 0^{\circ }$.

Aside from φ, another parameter of interest in tandem wings is *L* [29,31,33,35,36,37]. Boschitsch et al. [35] conducted Particle Image Velocimetry (PIV) measurements on two flapping wings arranged in a straight line underwater. The results showed that the thrust and thrust efficiency generated by the upstream wing differed from that of a single wing only when the wing spacing was relatively close (L/c<1.5). The aerodynamic performance of the downstream wing is mainly influenced by φ and *L*. By changing these two parameters, the thrust and thrust efficiency of the downstream wing can be adjusted to be 0.5 to 1.5 times that of a single wing. The results by Lua et al. [29] indicate that as the phase difference φ changes, the optimal distance *L* for the hindwing to produce maximum thrust also varies. For example, by adjusting the phase difference, the distance *L* at which the hindwing achieves maximum thrust shifts within the range of 1.5c to 3c. The authors claimed that within a certain range of St, the isolated effect of changing *L* on the average thrust of the hindwing could be equivalently achieved by independently changing φ. They proposed an equivalent relationship between *L* and φ. Recent papers on the coordinated motions of fish schools and bird formations further elucidate the interactions between the wake of the forewing and the hindwing [36,38,39]. For example, Joshi et al. [39], based on the direction of vortices in the wake of the forewing and their relative positions with respect to the hindwing, summarized five wake–wing interaction modes that either favor or hinder thrust development. They further proposed different flapping modes for tandem wings based on these interactions.

From the above, it can be inferred that existing research has revealed the primary effects of phase difference (φ) and wing spacing (*L*) on the aerodynamic characteristics of tandem wings. It is widely believed that wake–wing interactions are the primary factors determining the aerodynamic performance of tandem flapping wings.

Existing studies on tandem wing configurations often assume that the wings follow straight trajectories without surging motion. However, the actual flight trajectories of insects exhibit various complex patterns. For example, Fry et al. [40] found that fruit flies exhibit U-shaped trajectories during hovering. Willmott and Ellington [41] observed elliptical and figure-eight wingtip trajectories in freely flying hawkmoths. Wakeling and Ellington [42] observed that both the forewings and hindwings of freely flying dragonflies exhibit elliptical and figure-eight trajectories. Furthermore, Chen et al. [43] indicated that dragonfly hindwings can also exhibit double figure-eight trajectories. Many studies [9,20,44,45,46,47,48] on single wings indicate that the flapping trajectory can significantly alter their aerodynamic characteristics. Amiralaei et al. [46] conducted simulations on a thin ellipsoidal two-dimensional airfoil with a figure-of-eight trajectory. The results indicate that various trajectory parameters can quantitatively and qualitatively alter the instantaneous force coefficients. Esfahani et al. [47] investigated the effects of elliptical motion trajectory on a flapping wing. The results indicate that the motion trajectory simultaneously changes the effective angle of attack, as well as the vortex motion patterns in the flow field. Consequently, these changes significantly affect the aerodynamic and propulsive performance. Yang et al. [48] conducted numerical simulations on a two-dimensional airfoil with elliptical, figure-eight, and double figure-eight trajectories of different amplitudes. The results indicate that the figure-eight trajectory can enhance thrust and thrust efficiency. Although these studies were conducted under different conditions, some general patterns can still be summarized: Under certain conditions, the mean thrust of elliptical and figure-eight trajectories can be higher than the thrust of straight trajectories [46,47,48], while the mean thrust of double figure-eight trajectories is almost always lower than the thrust of straight trajectories [48]; The thrust efficiency of elliptical and double figure-eight trajectories is close to or lower than the efficiency of straight trajectories, whereas under certain conditions the thrust efficiency of figure-eight trajectories can exceed the efficiency of straight trajectories [48]; The vorticity evolution trend of elliptical trajectories is similar to straight trajectories [47], while figure-eight and double figure-eight trajectories exhibit significant multiple vortices [48].

However, in tandem wing configurations, there has been limited research focusing on the influence of surging motions of the hindwing on the evolution of the flow field. To fill these gaps, this study aims to investigate the effects of phase difference and surging motions of the hindwing on the aerodynamics of two-dimensional flapping wings in the forward flight mode. The rest of the paper is organized as follows. The motion of the tandem wings and the corresponding numerical methods are introduced in Section 2 and Section 3, respectively. The influence of phase difference and hindwing trajectory on the aerodynamics and flow field of tandem wing configurations are extensively detailed in Section 4. Subsequent Section 5 summarizes the conclusions and limitations of the present study and proposes future prospects.

## 2. Problem Formulation

**Figure 1:**
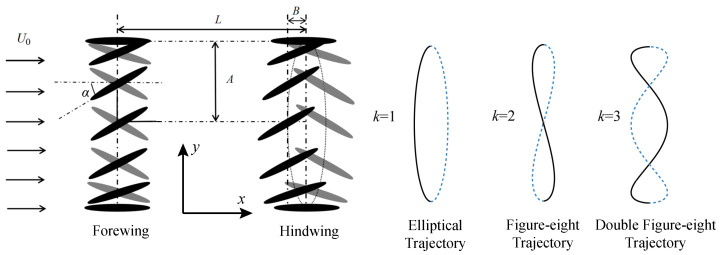
Schematic of the tandem wing configurations under consideration. The hindwing adopts an elliptical trajectory with $B_{m}>0$. Black ellipses represent downstroke positions, and grey ones denote upstroke positions. In the panels for the trajectories, if $B_{m}>0$, then solid lines indicate the downstroke, and dashed lines indicate the upstroke. If $B_{m}<0$, then dashed lines represent the downstroke, and solid lines represent the upstroke.

The tandem wing configuration used in the present calculations is depicted in Figure 1. Similar to the previous studies [29,31,37] on 2D tandem wings, simple sinusoidal functions are employed to control the vertical motion and rotation of the forewing. In addition to rotation and vertical motion, surging motion is introduced into the motion function of the hindwing. By adjusting the surging motion, the hindwing can exhibit various trajectories.

The motion of the forewing can be represented by the following equations:

(1) $$\begin{array}{cc} \; & y_{F}\left(t\right)=A\cos\left(2\pi ft\right) \end{array}$$

(2) $$\begin{array}{cc} \; & \alpha_{F}\left(t\right)=\alpha_{m}\sin\left(2\pi ft+\pi /2\right) \end{array}$$

where *t* is dimensional time, $y_{F}$, $\alpha_{F}$, *A*, *f* and $\alpha_{m}$ are the forewing heaving motion, forewing pitching motion, heaving amplitude, oscillating frequency, and pitching amplitude, respectively.

The motion of the hindwing can be represented by the following equations:

(3) $$\begin{array}{cc} \; & y_{H}\left(t\right)=A\cos\left(2\pi ft+\varphi \right) \end{array}$$

(4) $$\begin{array}{cc} \; & x_{H}\left(t\right)=B_{m}\sin\left(2\pi kft+k\varphi \right) \end{array}$$

(5) $$\begin{array}{cc} \; & \alpha_{H}\left(t\right)=\alpha_{m}\sin\left(2\pi ft+\pi /2+\varphi \right) \end{array}$$

where $y_{H}$, $x_{H}$, $\alpha_{H}$, and φ are hindwing heaving motion, hindwing surging motion, hindwing pitching motion, and the phase angle between the forewing and hindwing kinematics, respectively. $B_{m}$ is the amplitude of the surging motion, and *k* is the frequency. By tuning k=1,2and3, hindwing will display elliptical trajectory, figure-eight trajectory, and double figure-eight trajectory, respectively, as shown in Figure 1. In all cases, *A*, *f*, and $\alpha_{m}$ are fixed at 0.75c (*c* is the wing chord length), 0.67 Hz, and $30^{\circ }$, respectively. The selection of $\alpha_{m}$ is based on kinematic measurements of the dragonfly flight by Chen et al. [43]. *L* is the center-to-center wing spacing. According to Boschitsch et al. [35], the interaction between the hindwing and the forewing wake can be observed when L=1.5−2.5c. Additionally, according to Lua et al. [29], when L=1.5−4c, there exists a mathematical model that quantifies the L−φ relationship. Therefore, we fixed L=2.5c and varied the phase difference φ to analyze the interaction between the hindwing and the forewing wake. The freestream velocity $U_{0}$ is maintained at 0.125 m/s. The Reynolds number Re, based on *c* and $U_{0}$, is set at 2000, which corresponds to the range of dragonfly flight [49,50]. The resultant Strouhal number, $St=2fA/U_{0}$, is 0.32, corresponding to the range of reverse von Karman vortex streets where most animals generate thrust [15]. In the present study, to intuitively show the differences in the flow field, all figures and tables are plotted with the hindwing’s initial phase $y_{H}\left(t\right)=A$ at t/T=0.

The mean lift coefficient $C_{Lm}$ and mean thrust coefficient $C_{Tm}$ can be considered to be preliminary indicators of the aerodynamic performance of the flapping wings. They are defined as follows:

(6) $$\begin{array}{cc} \; & C_{Lm}=\frac{\frac{1}{T}\int_{t}^{t+T}F_{y}\left(t\right)dt}{\frac{1}{2}\rho c{U_{0}}^{2}} \end{array}$$

(7) $$\begin{array}{cc} \; & C_{Tm}=-\frac{\frac{1}{T}\int_{t}^{t+T}F_{x}\left(t\right)dt}{\frac{1}{2}\rho c{U_{0}}^{2}} \end{array}$$

where $F_{y}\left(t\right)$ and $F_{x}\left(t\right)$ are the instantaneous force in the positive *y* direction and *x* direction, respectively, ρ is the density of air. $C_{L}$ and $C_{T}$ represent the instantaneous lift coefficient and instantaneous thrust coefficient of the flapping wings, respectively. They are defined as the following equations:

(8) $$\begin{array}{cc} \; & C_{L}=\frac{F_{y}\left(t\right)}{\frac{1}{2}\rho c{U_{0}}^{2}} \end{array}$$

(9) $$\begin{array}{cc} \; & C_{T}=-\frac{F_{x}\left(t\right)}{\frac{1}{2}\rho c{U_{0}}^{2}} \end{array}$$

## 3. Numerical Method

The tandem wing simulation was carried out using the commercial computational fluid dynamics (CFD) package ANSYS FLUENT 2020 R2, solving the 2D unsteady, incompressible Navier–Stokes equations based on a finite volume method. The accuracy of this method has been extensively validated in relevant experiments and numerical studies [29,51,52]. Settings for some important solver parameters are as follows: A coupled scheme was employed for pressure-velocity coupling; A second-order upwind scheme was employed to solve the momentum equation, while temporal discretization was achieved through a first-order implicit formulation; The standard scheme was utilized for pressure discretization; According to Broering and Lian [53], the laminar model was applied to computations at a Reynolds number of 2000 in the present study.

**Figure 2:**
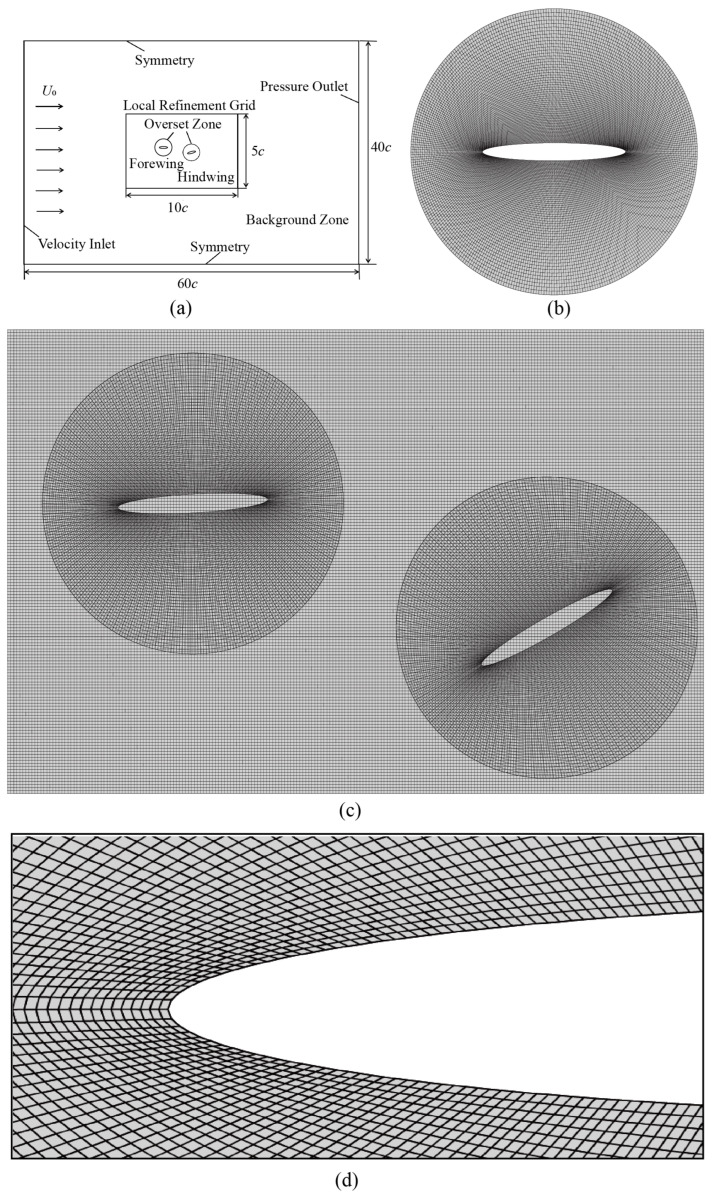
(**a**) Computational domain and boundary condition; (**b**) Foreground mesh used in the current calculation, with its density based on the results of the Mesh 2 grid convergence test in Table 1; (**c**) Local Refinement Grid Zone; (**d**) Mesh near the wing, with the first layer grid thickness approximately 0.0045*c*, resulting in a $y^{+}$ value of approximately 1.105.

The computational domain and boundary conditions for this study are schematically shown in Figure 2. A rectangular background grid with dimensions of 60c×40c is considered, in which two circular overset zones envelop the forewing and hindwing. The rigid body motion of the wings is achieved through the overset method and is controlled by the DEFINE ZONE MOTION command within the User Defined Function (UDF). As for the boundary conditions, the solid surfaces of the wings are set as no-slip walls. The left boundary of the computational domain is specified as a velocity inlet, the right boundary as a constant zero-gauge pressure, and symmetrical boundary conditions are applied to the top and bottom of the computational domain.

The structured foreground grid used in the simulations is depicted in Figure 2b. It is generated by projecting concentric ellipses onto a circle. Table 1 provides the results of the grid convergence test. In each test, the density of the foreground grid is adjusted, and the density of the local refinement zone in the background grid is adjusted to be roughly equal to that of the foreground grid region. Compared with Mesh 4, both $\Delta C_{Lm}$ and $\Delta C_{Tm}$ of Mesh 2 and Mesh 3 are less than 1%. To reduce unnecessary computational load, we employed the Mesh 2 grid in this study. Table 2 displays the outcomes of the time step independence test. Using the criteria that the $\Delta C_{Lm}$ and $\Delta C_{Tm}$ relative to case 4 are both less than 1%, the number of time steps per cycle is set to 1500.

To validate the above-defined numerical method, numerical simulations are carried out by calculating the typical cases available in the experimental results of Tuncer and Kaya [54] and the numerical results of Miao and Ho [55]. The validation model used a NACA0014 airfoil with a chord length of 0.1c. The trajectory of the airfoil motion was a straight trajectory, with the freestream velocity and angle of attack kept constant. The flapping amplitude *H* was 0.4c, the Reynolds number Re was $1\times 10^{4}$, and the reduced frequency was 2.

**Figure 3:**
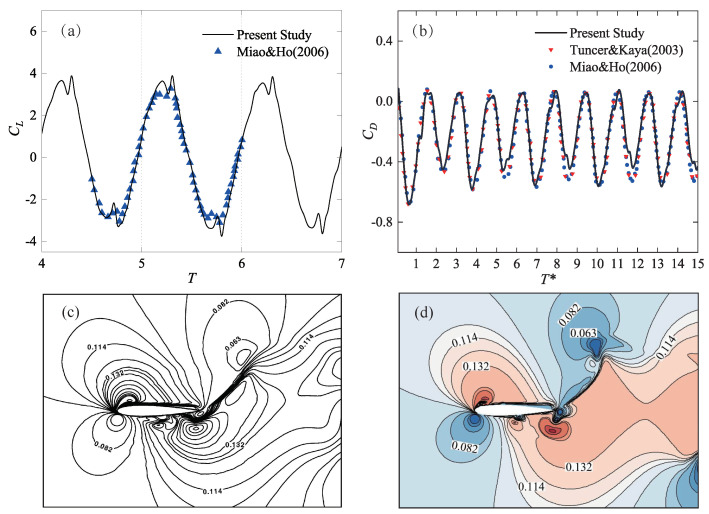
The validation results of forward flight conditions [54,55]. (**a**) Lift coefficient, *T* is the period; (**b**) Drag coefficient, $T^{*}$ is the nondimensional time, according to Miao and Ho [55]; (**c**) Mach number distribution cloud map by Miao and Ho [55]; (**d**) Mach number distribution cloud map by the present study.

Figure 3a,b compare the instantaneous aerodynamic force coefficients calculated in this study with the experimental results of Tuncer and Kaya [54] and the numerical results of Miao and Ho [55]. The results show very good agreement, with only minor differences at the peak values of $C_{L}$. Figure 3c,d compare the Mach number distribution cloud maps from this study with those from Miao and Ho [55], showing close matches. These comparisons indicate that the numerical method used in this study can accurately capture the aerodynamic performance of flapping wings.

## 4. Results and Discussions

In this section, we discuss the numerical results. In all calculations, *L* is fixed at 2.5c and St=0.32. Under this configuration, the motion of the hindwing has minimal impact on the flow field around the forewing [29,35]. In our simulations, changing the trajectory of the hindwing leads to only approximately 1% variations in the mean thrust coefficient of the forewing $C_{Tm\_f}$. This section focuses on the wake–wing interactions between the hindwing and the forewing wake. Section 4.1 will illustrate the effects of phase difference and surging motion on the mean thrust of the hindwing. Subsequent Section 4.2 and Section 4.3 will analyze these effects from the perspective of the flow field, first analyzing the flow field of a single wing with a straight trajectory, then introducing conditions such as the forewing, phase difference, and surging motion into the flow field.

### 4.1. Statistics of Hindwing Thrust

**Figure 4:**
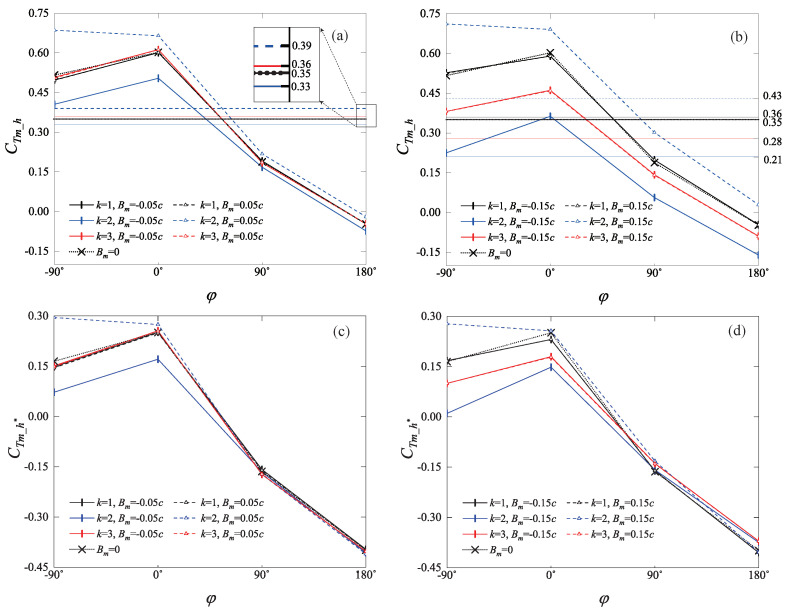
Variation of the force coefficients for the hindwing with phase differences for different trajectories. The trajectory of the forewing is fixed, and that of the hindwing varies, as shown in the legend. $C_{Tm\_h}$ represents the mean thrust coefficient of the hindwing. $C_{Tm\_h*}$ represents the increase in the mean thrust coefficient of the hindwing compared to a single wing with the same trajectory. It is calculated by subtracting the mean thrust coefficient of the single wing ($C_{Tm}$) from $C_{Tm\_h}$. (**a**) $C_{Tm\_h}$ vs. φ, $B_{m}=\left|0.05c\right|$. In the figure, horizontal lines without symbols represent the time-averaged thrust of a single wing with the same trajectory. Due to symmetry, the time-averaged thrust of the trajectories with $B_{m}=-0.05c$ and $B_{m}=+0.05c$ are the same for k=1 or 3; (**b**) $C_{Tm\_h}$ vs. φ, $B_{m}=\left|0.15c\right|$. Similar to (**a**), horizontal lines without symbols represent the time-averaged thrust of a single wing with the same trajectory; (**c**) $C_{Tm\_h*}$ vs. φ, $B_{m}=\left|0.05c\right|$; (**d**) $C_{Tm\_h*}$ vs. φ, $B_{m}=\left|0.15c\right|$.

Figure 4 illustrates the variation in the mean thrust $C_{Tm\_h}$ and the increase in mean thrust $C_{Tm\_h*}$ for the hindwing under different phase differences φ and surging motions ($B_{m},k$). We first focus on the straight trajectory, which shows the highest thrust coefficient at φ=0, followed by $-90^{\circ }$, $90^{\circ }$, and $180^{\circ }$. Also, the straight trajectory achieves the maximum mean thrust augmentation $C_{Tm\_h*}$ when φ=0, followed by $\varphi =-90^{\circ }$, while at $\varphi =90^{\circ }$ and $180^{\circ }$ the straight trajectory reduces the thrust of the hindwing. Especially at $\varphi =180^{\circ }$, the $C_{Tm\_h}$ of the straight trajectory is nearly zero. Similar trends are also observed in the studies of Lua et al. [29] and Muscutt et al. [31], indicating that phase differences alter the interactions between the hindwing and the wake of the forewing. The interaction modes resulting from the phase differences φ in this study will be discussed in detail in Section 4.2.

Next, we focus on the changes in the mean thrust of a single wing caused by the surging motion for k=1, 2, 3. In Figure 4a, the horizontal lines without symbols indicate the mean thrust of a single wing with the same trajectory as the hindwing. Among these, the mean thrust of the figure-eight trajectory with $B_{m}>0$ is the highest, followed by the double figure-eight trajectory, which is slightly higher than the straight trajectory. In contrast, the mean thrust of the elliptical trajectory is almost consistent with that of the straight trajectory. In Figure 4b, observations show that as $B_{m}$ increases, the single wing is more significantly affected by the trajectory effect. The figure-eight trajectory with $B_{m}>0$ significantly enhances the mean thrust of the hindwing, while the mean thrust of the elliptical trajectory remains close to that of the straight trajectory. However, the elliptical trajectory with $B_{m}<0$ and the double figure-eight trajectory both lead to a significant reduction in the mean thrust. Yang et al. [48] conducted a 2D simulation with a NACA0012 airfoil at St=0.28 and showed a similar trend in trajectory effects on the mean thrust of a single wing, which corroborates the reliability of the results in this paper.

Following this, we focus on the influence of trajectory effects on the mean thrust of the hindwing. It can be observed in Figure 4a,b, regardless of small ($\left|B_{m}\right|=0.05c$) or large ($\left|B_{m}\right|=0.15c$) amplitude, that at different phase differences, the $C_{Tm\_h}$ of the elliptical trajectory are essentially consistent with that of the straight trajectory. In terms of $C_{Tm\_h*}$, the elliptical trajectories also maintain a global consistency with the straight trajectory, as shown in Figure 4c,d. The figure-eight trajectories with $B_{m}>0$ enhance $C_{Tm\_h}$ under all phase differences, and this effect is the most pronounced at $\varphi =-90^{\circ }$. However, at $B_{m}<0$, the figure-eight trajectories weaken $C_{Tm\_h}$; $C_{Tm\_h}$ of the double figure-eight trajectories are essentially the same as that of the straight trajectory under $\left|B_{m}\right|=0.05c$, while under $\left|B_{m}\right|=0.15c$, the double figure-eight trajectories reduce $C_{Tm\_h}$. Additionally, at $\left|B_{m}\right|=0.05c$, $C_{Tm\_h}$ and $C_{Tm\_h*}$ of each trajectory are very close to those of the straight trajectory. At $\left|B_{m}\right|=0.15c$, trajectories significantly alter the aerodynamic performance of the hindwing. In Figure 4d, with phase differences of −90° and 0°, the $C_{Tm\_h*}$ of the four trajectories show significant differences, indicating that under these conditions, adjusting the trajectory can significantly alter the thrust obtained by the hindwing from the wake–wing interaction. However, with phase differences of 90° and 180°, the $C_{Tm\_h*}$ of the four trajectories are almost identical, which means that in these cases, adjusting the trajectory of the hindwing does not significantly change the $C_{Tm\_h*}$.

The aforementioned phenomena can be attributed to two factors. First, the variation of hindwing trajectory induces changes in its aerodynamic performance, which has been extensively discussed in the existing single-wing studies [9,20,44,45,46,47,48]; Second, the alteration in the hindwing surging motions affects its interaction with the wake flow generated by the forewing, a subject that will be discussed in detail in Section 4.3.

Another notable observation in Figure 4 is the symmetry of $C_{Tm\_h}$ and $C_{Tm\_h*}$. If two cases have the same *k* (both are 1 or both are 3), and $B_{m}$ is opposite (for example, one is +0.15c and the other is −0.15c), then $C_{Tm\_h}$ and $C_{Tm\_h*}$ for these two cases will be the same. A simple approach illustrating this symmetry will be presented in Appendix A.

### 4.2. Phase Difference

**Figure 5:**
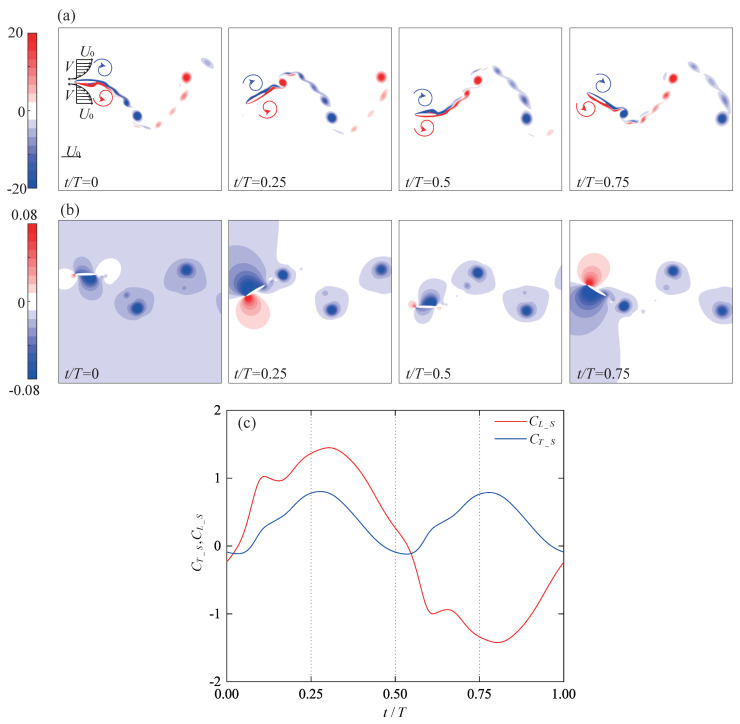
Flow field and aerodynamics of single wing with straight trajectory (**a**) Vorticity clouds for single wing; (**b**) Pressure clouds for single wing; (**c**) Instantaneous aerodynamic force for single wing.

To begin with, a single wing with a straight trajectory ($B_{m}=0$) is employed to illustrate the evolution of the flow field and aerodynamics within one flapping cycle. As shown in Figure 5a, during the forward flight, the flow within the boundary layer on the surfaces of the wing is attached and follows the wing’s movement, while the flow away from the surfaces can be considered to move at the incoming flow velocity. This results in velocity gradients both above and below the wing surface. During the downward stroke, as shown in Figure 5b, clockwise vortices induce suction pressure regions on the upper surface of the wing. The shape of the suction pressure region changes with the evolution of vortices on the upper surface. Simultaneously, the lower surface of the wing generates high-pressure regions influenced by the effective angle of attack. Therefore, the downstroke generates positive lift and positive thrust, as shown in Figure 5c. During the upstroke, the leading-edge vortices (LEV) primarily evolve on the lower surface, inducing the corresponding suction pressure regions. The upper surface, on the contrary, generates high-pressure regions. Therefore, this pressure difference results in positive thrust and negative lift.

**Figure 6:**
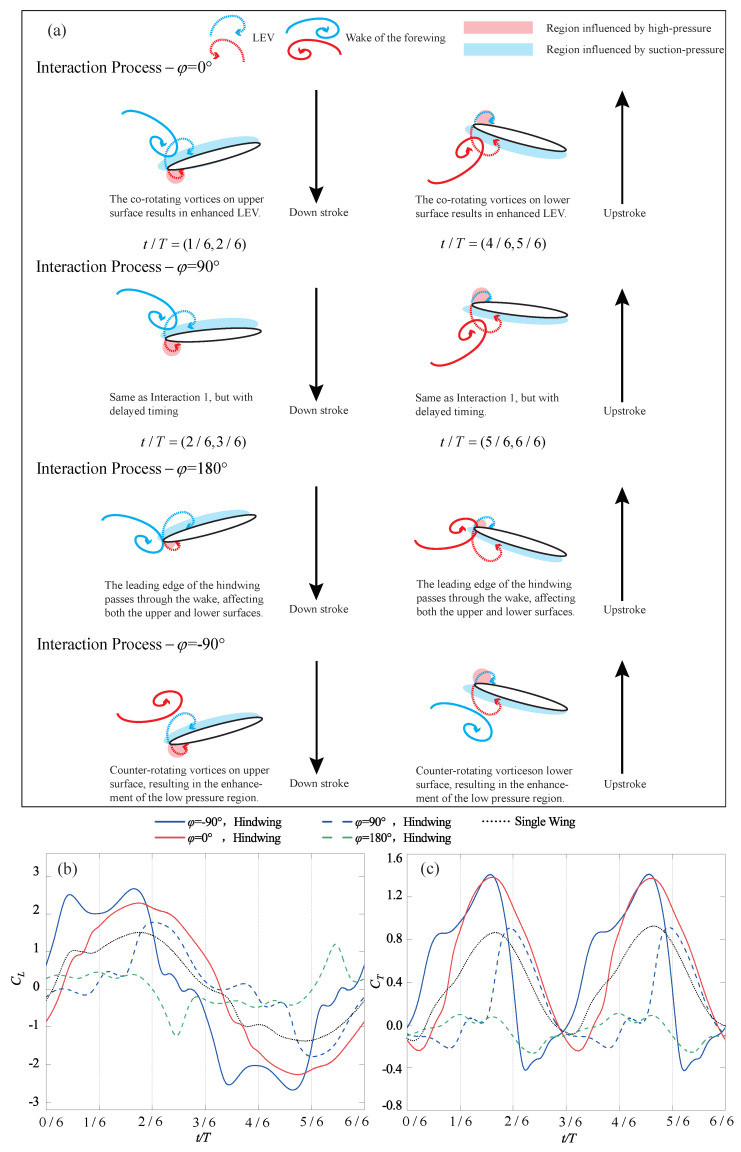
Schematic representation of four types of wake–wing interaction processes and their corresponding aerodynamics (**a**) Wake–wing interaction processes; (**b**) Instantaneous lift coefficient; (**c**) Instantaneous thrust coefficient.

**Figure 7:**
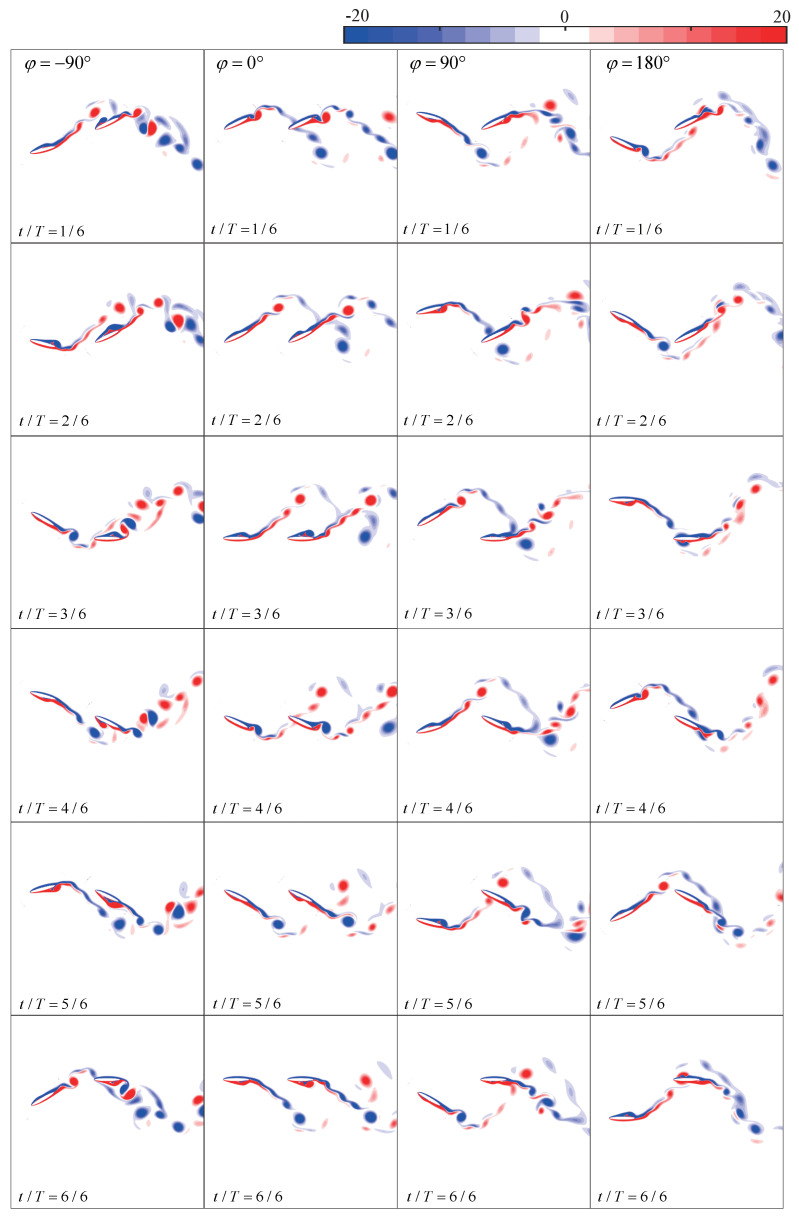
Vorticity cloud map for the four types of wake–wing interaction processes.

**Figure 8:**
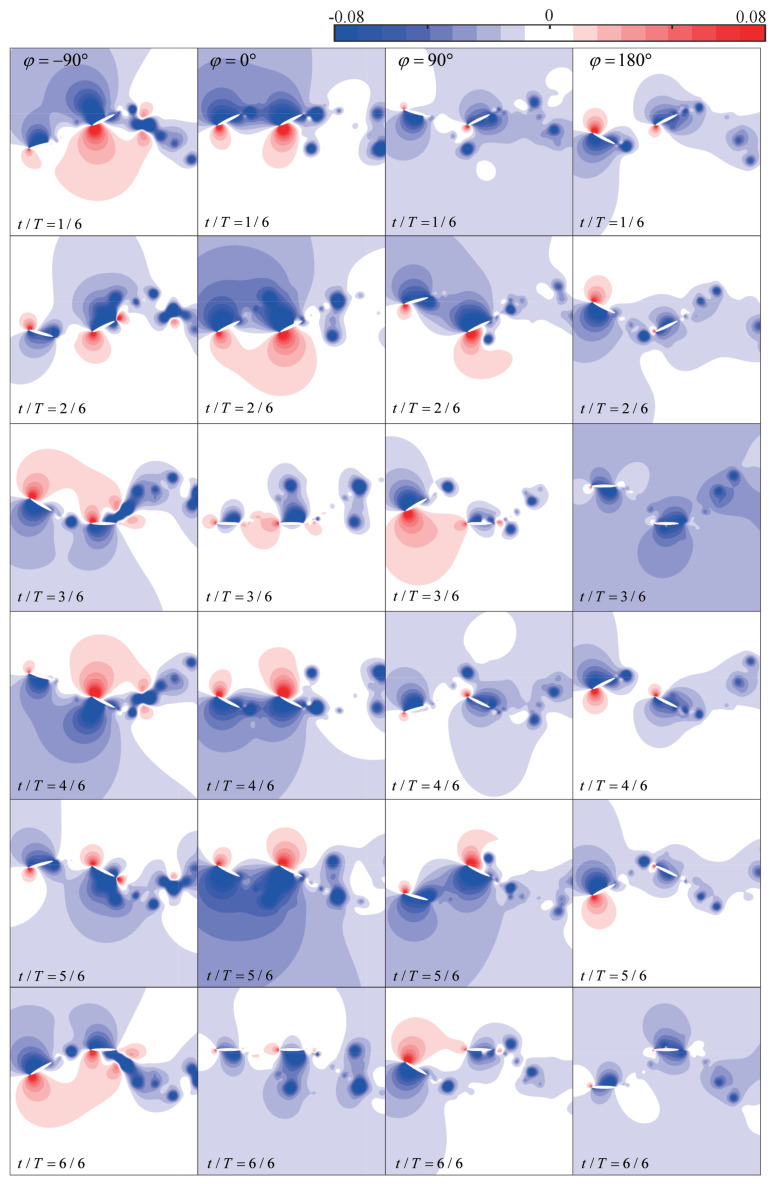
Pressure contour map for the four types of wake–wing interaction processes.

Furthermore, the same straight trajectory as in Figure 5 is introduced into the tandem flapping wing configuration, with the phase difference φ subsequently varied as $-90^{\circ }$, $0^{\circ }$, $90^{\circ }$, and $180^{\circ }$. Figure 6 illustrates the wake–wing interaction processes corresponding to these four phase differences, along with the corresponding instantaneous aerodynamic coefficients (their time-averaged aerodynamic coefficients are shown in Figure 4). Figure 7 and Figure 8 represent the corresponding vorticity and pressure distributions, respectively.

In the case of $\varphi =0^{\circ }$, during the downstroke, the upper surface of the hindwing sweeps over the co-rotating wake vortices of the forewing (around t/T=(1/6, 2/6)), increasing the accumulation of vorticity on the upper surface. This induces a larger suction pressure region compared to the single-wing flow field and enhances the upwash high-pressure region on the lower surface, as shown in Figure 8. Therefore, in Figure 6b,c, the aerodynamics generated by this wake–wing interaction model are superior to the single-wing control group during the downstroke process (around t/T=(1/6, 3/6)). These thrust-enhancing wake–wing interaction mechanisms have been observed in the work of Lua et al. [29] and Joshi et al. [39], validating the reliability of the results presented in this paper. Similarly, during the upstroke process, the lower surface of the hindwing receives co-rotating vorticity from the forewing (around t/T=(4/6, 5/6)), increasing the pressure difference and therefore enhancing overall aerodynamic force. However, at this point, the overall aerodynamic force is characterized by negative lift and positive thrust.

$\varphi =90^{\circ }$: Similar to $\varphi =0^{\circ }$, during the downstroke, the upper surface of the hindwing also sweeps across co-rotating wake vortices (around t/T=(2/6, 3/6)), resulting in increases in aerodynamic forces. However, the accumulated vortices on the upper surface cannot completely detach at the end of the downstroke. The remaining vortices continue to induce suction pressure regions during the upstroke (at t/T=(2/6, 3/6)), resulting in deteriorations in the aerodynamics of the hindwing. Similarly, at the end of the upstroke, vortices accumulate on the lower surface of the hindwing. This leads to deteriorations in the aerodynamic performance during the first half of the subsequent downstroke, as shown in Figure 6b,c. Thus, despite the wake–wing interaction mechanism being the same as $\varphi =0^{\circ }$, the average thrust still differs by a factor of four due to the delayed timing of vorticity input.

$\varphi =180^{\circ }$: At the beginning of the downstroke, the lower surface of the hindwing sweeps through the wake, creating larger suction pressure regions at the trailing edge. Subsequently, as the leading edge of the hindwing passes through the wake vortex, it induces suction pressure regions by causing vortices to attach to the lower surface of the hindwing. Simultaneously, this alters the instantaneous angle of attack of the hindwing, resulting in the formation of a high-pressure region on the upper surface. Both effects contribute to the deterioration of the overall aerodynamic force of the hindwing. A similar situation occurs during the upstroke, where this wake–wing interaction leads to severe degradations of the aerodynamic force, as depicted in Figure 6b,c. Joshi et al. [39] summarized the similar wake–wing interaction mechanisms. However, due to the differences in the timing of vorticity interaction and the configuration of the foil, this mechanism in their study led to a favorable condition for thrust generation.

$\varphi =-90^{\circ }$: In the downstroke, the counter-rotating wake vortices pass over the upper surface of the hindwing. Although these vortices do not adhere to the upper surface, they can still enhance the suction pressure region on the upper surface. Subsequently, the hindwing traverses the weaker portion of the wake, similar to the case of $\varphi =180^{\circ }$. This results in a transient deterioration in aerodynamic performance (around t/T=(2/6, 3/6)). During the upstroke, the lower surface of the hindwing also experiences aerodynamic enhancement from the counter-rotating wake vortices. Unlike the case of $\varphi =90^{\circ }$, the counter-rotating vortices, in this case, do not adhere to the wing surface. Consequently, they do not diminish the aerodynamic performance during the subsequent downstroke. The mechanism of the counterclockwise vortex flowing near the upper surface of the foil, enhancing the suction pressure, has also been pointed out in the work of Joshi et al. [39].

Through the aforementioned four wake–wing interaction processes, some patterns can be summarized. If the wake vortices of the forewing appear on the surface of the hindwing that generates suction pressure regions, i.e., the upper surface during the downstroke and the lower surface during the upstroke, it leads to increases in the instantaneous aerodynamic forces. Joshi et al. [39] obtained similar conclusions by varying the chord and gap ratios of the hindwing. Conversely, if they appear in the region where the hindwing generates high-pressure regions, i.e., the lower surface during the downstroke and the upper surface during the upstroke, it leads to a deterioration in the aerodynamic forces. Additionally, if the vortices inducing suction pressure regions cannot detach promptly at the end of the flapping cycle, they will decrease the aerodynamic forces in the initial phase of the subsequent cycle.

### 4.3. Surging Motion

#### 4.3.1. Elliptical Trajectory

**Figure 9:**
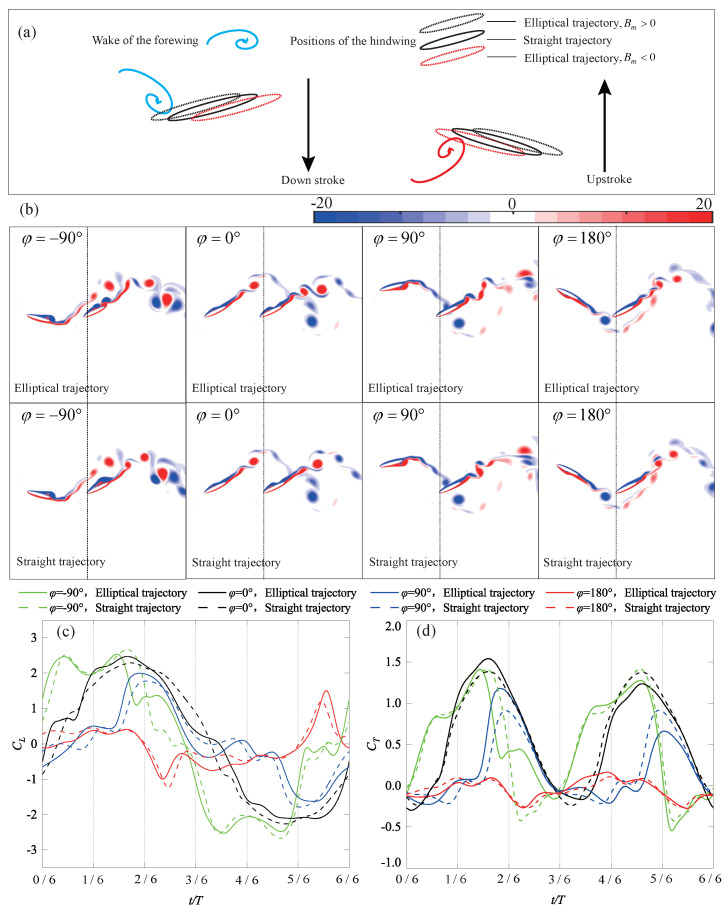
Schematic representation of the elliptical trajectory of wake–wing interactions and their corresponding aerodynamics (**a**) Wake–wing interactions; (**b**) Flow field comparison between the elliptical trajectory ($B_{m}=0.15c$, t/T=2/6) and the straight trajectory; (**c**) Instantaneous lift coefficient ($B_{m}=0.15c$); (**d**) Instantaneous thrust coefficient ($B_{m}=0.15c$).

As shown in Figure 9a, elliptical trajectories with $B_{m}>0$ cause the hindwing to encounter the forewing vortex street earlier during the downstroke and later during the upstroke. Although wake–wing interactions remain the same as the straight trajectory, their timing is accordingly advanced or delayed, as shown in Figure 9a,b. For $\varphi =0^{\circ }$ and $90^{\circ }$, the advanced interaction causes the vortices contributing to the increased aerodynamics to attach to the wing surface earlier, resulting in an aerodynamic enhancement. Conversely, the delayed interaction has the opposite effect. Therefore, in Figure 9c,d, the aerodynamics during the downstroke of the elliptical trajectory will be higher than that of the straight trajectory, while the aerodynamic force during the upstroke will be lower than the straight trajectory. However, for $\varphi =-90^{\circ }$ and $180^{\circ }$, since vortices from the forewing do not significantly attach to the hindwing, the surging motion only changes the timing and magnitude of the aerodynamic peak.

#### 4.3.2. Figure-Eight Trajectory

**Figure 10:**
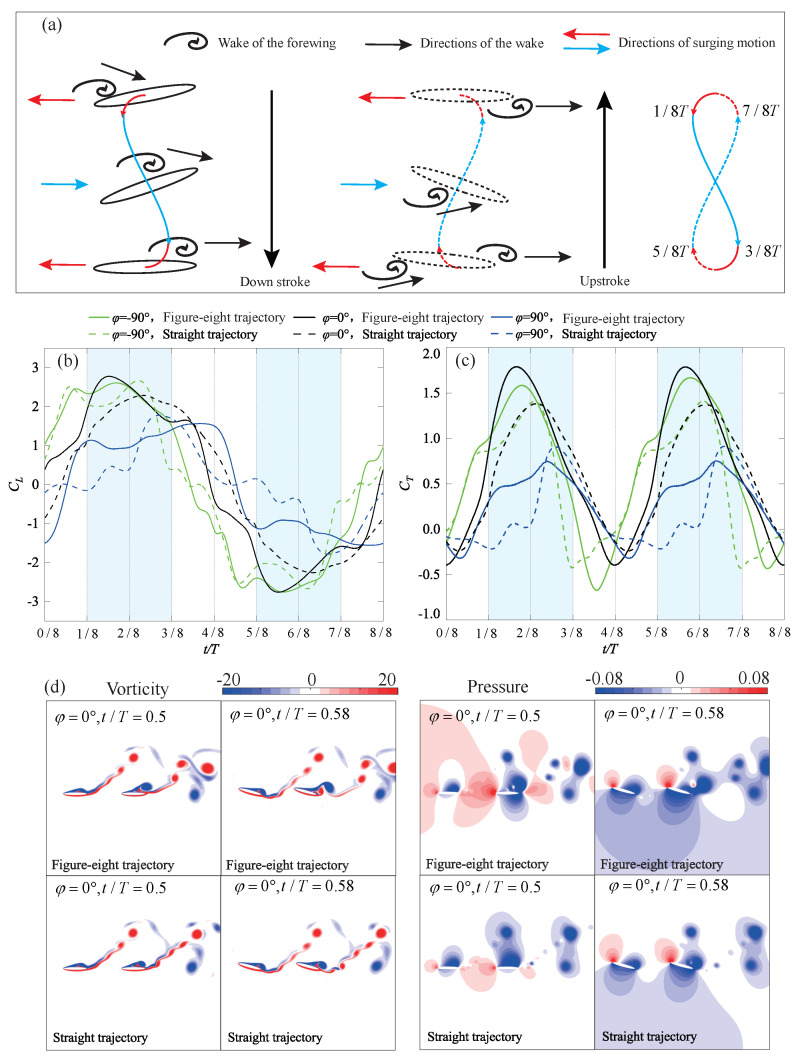
Schematic representation of the figure-eight trajectory of wake–wing interactions and their corresponding aerodynamics (**a**) Wake–wing interactions; (**b**) Instantaneous lift coefficient ($B_{m}=0.15c$); (**c**) Instantaneous thrust coefficient ($B_{m}=0.15c$); (**d**) Comparison of flow field details.

It can be observed from Figure 4 that for phase differences of $0^{\circ }$ and $-90^{\circ }$, the figure-eight trajectory, with $B_{m}>0$, significantly enhances the aerodynamic thrust of the hindwing. However, for phase differences of $90^{\circ }$ and $180^{\circ }$, the $C_{Tm\_h*}$ of the figure-eight trajectory is essentially the same as that of the straight trajectory. Figure 10a further explains the mechanism behind these observations under the phase differences of $-90^{\circ }$ and $0^{\circ }$. During the period from 0 to 1/8 *T*, the forward surging motion facilitates the early contact of the hindwing with the wake of the forewing. Between 1/8 *T* and 3/8 *T*, the direction of surging motion aligns with the motion of vortices on the upper surface of the hindwing. This aids in keeping the vortices attached to the upper surface, inducing a larger suction pressure region. For most of this period, the aerodynamic thrust generated by the figure-eight trajectory exceeds that of the straight trajectory, as illustrated in Figure 10c. During the period from 3/8 *T* to 5/8 *T*, the direction of surging motion returns to the forward direction, facilitating the detachment of vortices from the trailing edge of the upper surface of the hindwing. However, during this phase, the aerodynamics is lower than that of the straight trajectory. However, this prevents the vortices from accumulating on the upper surface of the hindwing, allowing the hindwing to experience a rapid thrust increase at the beginning of the upstroke (4/8 *T*–5/8 *T*). Figure 10d illustrates the flow details of this process. Compared to the straight trajectory, the figure-eight trajectory accumulates more vortices on the upper surface at the end of the downstroke (0.5 *T*), and these vortices detach more rapidly from the trailing edge. As shown in Figure 10a, the vortex evolution during the upstroke mirrors that of the downstroke, yet it results in negative lift and positive thrust.

In summary, throughout one flapping cycle, the figure-eight trajectory both enhances and maintains vortices that are beneficial for thrust generation. At the end of the flapping cycle, it facilitates the prompt shedding of these vortices, therefore preventing a deterioration in aerodynamic forces at the beginning of the next cycle. This trajectory can enhance the hindwing thrust at phase differences of $-90^{\circ }$ and $0^{\circ }$. However, at phase differences of $90^{\circ }$ and $180^{\circ }$, the figure-eight trajectory still fails to obtain thrust enhancement from the wake of the forewing.

#### 4.3.3. Double Figure-Eight Trajectory

**Figure 11:**
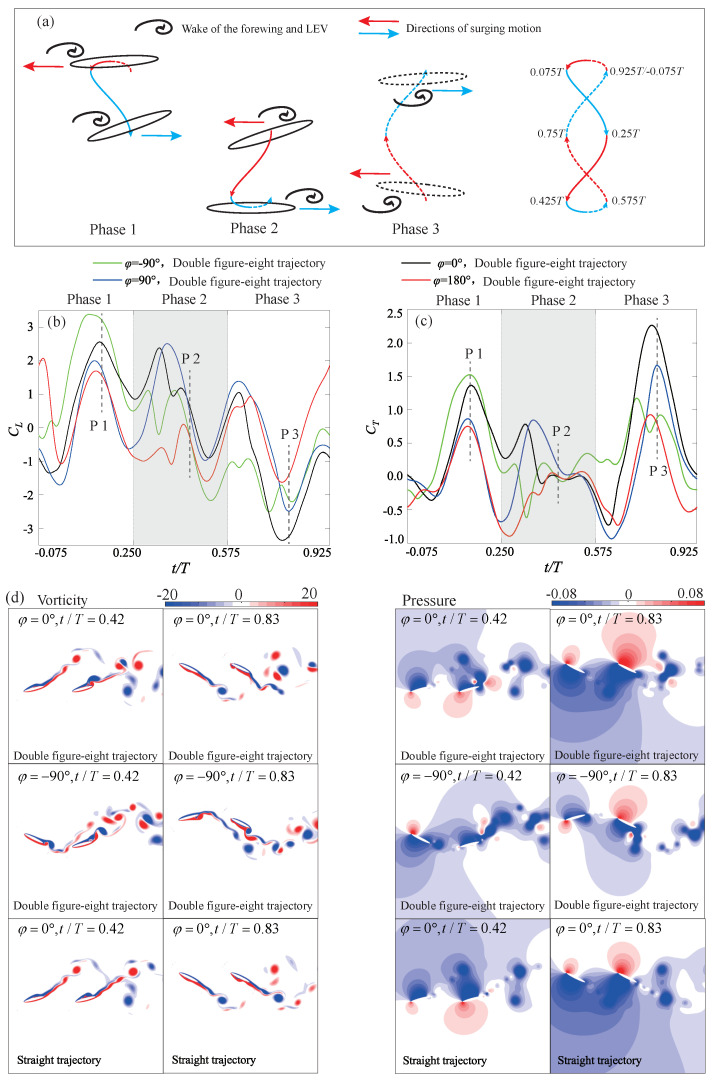
(**a**) Motion decomposition of the double-figure-eight trajectory; (**b**) Instantaneous lift coefficient ($B_{m}=0.15c$); (**c**) Instantaneous thrust coefficient ($B_{m}=0.15c$); (**d**) Comparison of flow field details ($B_{m}=0.15c$).

In Figure 4b, the average thrust generated by the double figure-eight trajectory is significantly lower than that of the straight trajectory across all four phase differences. Figure 11 elucidates the reasons behind the reduction in hindwing thrust caused by the double figure-eight trajectory. The motion of the double-figure-eight trajectory within one cycle can be decomposed into three phases, as illustrated in Figure 11a. Each phase is accompanied by one cycle of reciprocating surging motion. In Phase 1, forward surging motion causes the hindwing to make early contact with the wake of the forewing. Subsequently, the backward surging motion causes the vortices to accumulate on the upper surface of the hindwing. For the four phase differences considered, the curves all reach a peak in lift and thrust near the P1 moment. In the subsequent Phase 2, forward surging motion resumes, accelerating the shedding of vortices from the forewing and the LEV of the hindwing from the upper surface, as shown in Figure 11d, at t/T=0.42. As the shedding of vortices occurs earlier than the end of the downstroke, aerodynamic forces are significantly reduced in this phase. Therefore, on the one hand, a decrease in the pressure difference on both sides of the hindwing at t/T=0.42 can be observed in the pressure cloud diagrams of Figure 11d for the double figure-eight trajectory. On the other hand, a significant reduction in lift and thrust around the P2 moment is also observed in Figure 11b,c. In the subsequent Phase 3, the combination of forward and then backward surging motion is favorable for the wing to acquire vortices during the upstroke. Therefore, aerodynamic forces are significantly enhanced during this phase.

The three Phases are marked at specific time points within a flapping cycle in Figure 11a, indicating that the surging motion’s strokes are not synchronized with the downstroke and upstroke phases of the wing flapping, as well as with the phase of the incoming vortex street. Although the paths of the surging motion during the downstroke and upstroke are antisymmetric when k=3, their effects do not cancel each other out. Consequently, the double figure-eight trajectory does not exhibit the antisymmetric flow field evolution seen in the straight trajectory, as illustrated in Figure 11d.

In summary, the double figure-eight trajectory also significantly alters the interaction between the hindwing and the wake of the forewing. However, due to the premature shedding of vortices in Phase 2, the overall thrust of this trajectory is lower performance compared to a straight trajectory or an elliptical trajectory.

## 5. Conclusions

This study investigates the influence of the hindwing trajectory on the aerodynamic performance of tandem flapping wing configurations under the forward flight conditions at Re=2000 and St=0.32. It is revealed that, although the aerodynamic performance of the hindwing is primarily influenced by the phase difference φ, specific surging motions can still result in the enhancement or reduction of the hindwing’s thrust.

By analyzing wake–wing interaction processes corresponding to $\varphi =-90^{\circ },\;0^{\circ },\;90^{\circ }\;$ and $\;180^{\circ }$ the following evolution behaviors are summarized. If the wake vortices of the forewing appear on the side surface of the hindwing that generates low pressure, i.e., the upper surface during the downstroke and the lower surface during the upstroke, it leads to increases in the instantaneous aerodynamic forces; conversely, if they appear in the region where the hindwing generates high pressure, i.e., the lower surface during the downstroke and the upper surface during the upstroke, it leads to a deterioration in the aerodynamic performance of the hindwing. Additionally, if the vortices inducing suction pressure regions cannot detach promptly at the end of the flapping cycle, they will decrease the aerodynamic performance in the initial phase of the subsequent cycle. Based on the observed trends, $\varphi =-90^{\circ }$ and $0^{\circ }$ contribute to aerodynamics enhancement for the hindwing from the wake of the forewing, while $\varphi =90^{\circ }\;$ and $180^{\circ }$ phase differences lead to reductions in aerodynamic performance.

The elliptical trajectory does not have a significant impact on the aerodynamic performance of the hindwing, while the figure-eight trajectory with $B_{m}>0$ can significantly enhance the thrust of the hindwing. However, the double figure-eight trajectory tends to decrease the thrust of the hindwing. Because the evolution of vortices corresponding to surging motion with k=1 remains unchanged, surging motion with k=2 favors the hindwing in obtaining aerodynamic thrust, while surging motion with k=3 leads to premature vortex shedding, resulting in insufficient aerodynamic force.

In the end, it should be noted that the present study did not consider factors such as wing distance, heaving amplitudes, wing stiffness, and their impact on the coupling effects in trajectory analysis. Additionally, this study lacks support for 3D simulations and experimental data. In the future, we plan to extend this research by incorporating parameters such as flapping amplitude and wing distance. We also aim to compare 3D flow field tracing experiments with 2D simulations to determine the applicability of the 2D trajectory effect simulation results to 3D motions

## Figures and Tables

**Table 1:** Grid convergence test.

Number	Number of Cells in the Foreground Grid	$C_{Lm}$	$\Delta C_{Lm}$	$C_{Tm}$	$\Delta C_{Tm}$
Mesh 1	3000	1.546	−2.08%	1.224	−2.21%
Mesh 2	12,000	1.580	0.07%	1.242	−0.76%
Mesh 3	27,000	1.574	−0.30%	1.242	−0.76%
Mesh 4	48,000	1.579	-	1.251	-

**Table 2:** Time step size independence test.

Number	Time Step Size	$C_{Lm}$	$\Delta C_{Lm}$	$C_{Tm}$	$\Delta C_{Tm}$
Case 1	T/500	1.559	−1.541%	1.219	−2.949%
Case 2	T/1000	1.564	−1.210%	1.235	−1.650%
Case 3	T/1500	1.573	−0.606%	1.246	−0.822%
Case 4	T/2000	1.583	-	1.256	-

**Figure A1:**
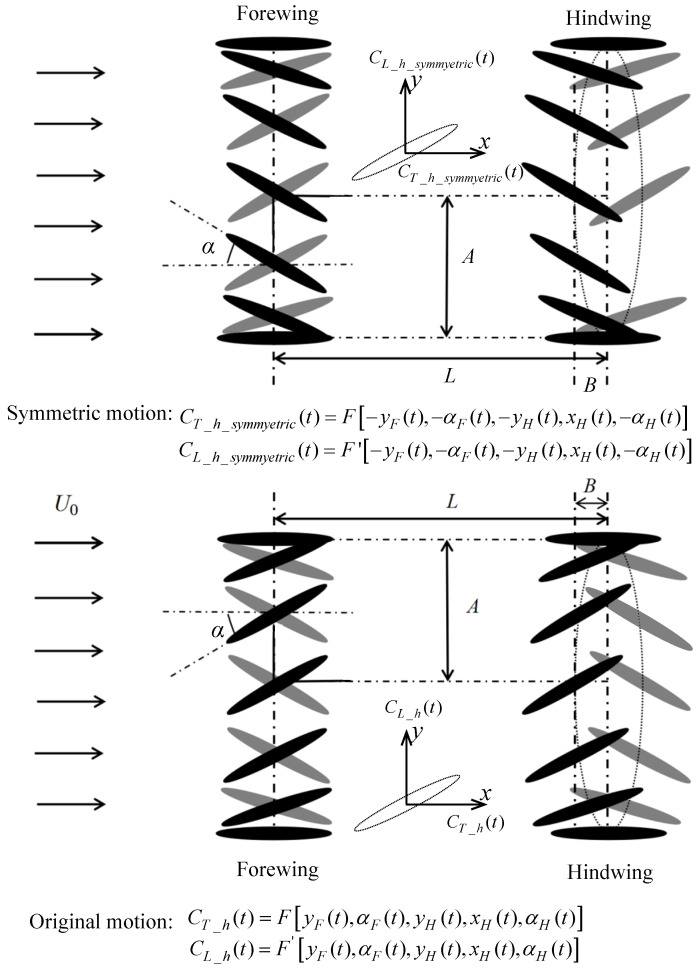
Schematic representation of the symmetry of the motion model.

## Data Availability

Data are contained within the article.

## Abbreviations

The following abbreviation is used in this manuscript:

LEV	Leading-Edge Vortices

## Appendix A

This section presents a simple method for proving the symmetry of 2D harmonic function trajectories.

1. When $U_{0}$, *L*, and φ are fixed, the thrust coefficient of the hindwing $C_{T\_h}$ and the lift coefficient of the hindwing $C_{L\_h}$ can be considered to be functions of $y_{F},\alpha_{F},y_{H},x_{H}$, and $\alpha_{H}$. Once the flow field reaches a quasi-steady state, it can be considered that:
  (A1) $$\begin{array}{cc} \; & C_{T\_h}\left(t\right)=F\left[y_{F}\left(t\right),\alpha_{F}\left(t\right),y_{H}\left(t\right),x_{H}\left(t\right),\alpha_{H}\left(t\right)\right] \end{array}$$
2. Due to the trigonometric properties of the motion trajectory, when *k* is odd, the following relationship exists:
  (A2) $$\begin{array}{cc} \; & C_{T\_h}\left(t+T/2\right)=F\left[-y_{F}\left(t\right),-\alpha_{F}\left(t\right),-y_{H}\left(t\right),-x_{H}\left(t\right),-\alpha_{H}\left(t\right)\right] \end{array}$$
3. Assuming there is a symmetric motion as shown in Figure A1, with flow field variations identical to the original motion but observed from a different perspective.
  (A3) $$\begin{array}{cc} \; & C_{T\_h}\left(t\right)={C_{T\_h}}_{\_symmyetric}\left(t\right) \end{array}$$
  (A4) $$\begin{array}{cc} \; & C_{T\_h}\left(t\right)=F\left[-y_{F}\left(t\right),-\alpha_{F}\left(t\right),-y_{H}\left(t\right),x_{H}\left(t\right),-\alpha_{H}\left(t\right)\right] \end{array}$$
4. Combining Equations (A2) and (A4):
  (A5) $$\begin{array}{cc} \; & C_{T\_h}\left(t+T/2\right)=F\left[y_{F}\left(t\right),\alpha_{F}\left(t\right),y_{H}\left(t\right),-x_{H}\left(t\right),\alpha_{H}\left(t\right)\right] \end{array}$$
  This means that trajectories with $+B_{m}$ and $-B_{m}$ actually have the same $C_{T\_h}$, differing only by half a period in phase. Therefore, the mean thrust coefficients are equal. Similarly, it can be concluded that their $C_{L\_h}$ satisfy that: $C_{L\_h\_+B_{m}}\left(t+T/2\right)=-C_{L\_h\_-B_{m}}\left(t\right)$. The conditions for the above relations to hold are that *k* is an odd number.

## References

[1] Alexander R. (2000). Form and function of insect wings: The evolution of biological structures. Nature.

[2] Shyy W., Lian Y., Tang J., Viieru D., Liu H. (2007). Aerodynamics of Low Reynolds Number Flyers.

[3] Vargas A., Mittal R., Dong H. (2008). A computational study of the aerodynamic performance of a dragonfly wing section in gliding flight. Bioinspir. Biomim..

[4] Lee S.H., Lahooti M., Kim D. (2018). Aerodynamic characteristics of unsteady gap flow in a bristled wing. Phys. Fluids.

[5] Cheng X., Sun M. (2021). Wing kinematics and aerodynamic forces in miniature insect Encarsia formosa in forward flight. Phys. Fluids.

[6] Zheng L., Hedrick T.L., Mittal R. (2013). Time-Varying Wing-Twist Improves Aerodynamic Efficiency of Forward Flight in Butterflies. PLoS ONE.

[7] Noda R., Liu X., Hefler C., Shyy W., Qiu H.H. (2023). The interplay of kinematics and aerodynamics in multiple flight modes of a dragonfly. J. Fluid Mech..

[8] Li C., Dong H. (2017). Wing kinematics measurement and aerodynamics of a dragonfly in turning flight. Bioinspir. Biomim..

[9] Xiao S., Hu K., Huang B., Deng H., Ding X. (2021). A Review of Research on the Mechanical Design of Hoverable Flapping Wing Micro-Air Vehicles. J. Bionic Eng..

[10] Hu Y., Ru W., Liu Q., Wang Z. (2022). Design and Aerodynamic Analysis of Dragonfly-like Flapping Wing Micro Air Vehicle. J. Bionic Eng..

[11] Zhang R., Zhang H., Xu L., Xie P., Wu J., Wang C. (2022). Mechanism and Kinematics for Flapping Wing Micro Air Vehicles Maneuvering Based on Bilateral Wings. Int. J. Aerosp. Eng..

[12] Zhang R., Hu W., Zheng X., Xu L., Wang C. (2022). Improvement of Flapping Mechanism and Measurement of Aerodynamic Force/Moment of Bionic Dragonfly Prototype. J. Aerosp. Power.

[13] Jia B.B., Gong W.Q. (2016). Force and Flow Field Measurement System for Tandem Flapping Wings. Exp. Tech..

[14] Sun X., Gong X., Huang D. (2017). A review on studies of the aerodynamics of different types of maneuvers in dragonflies. Arch. Appl. Mech..

[15] Guyon E., Hulin J.-P., Petit L. (2012). Hydrodynamique Physique.

[16] Taylor G.K., Nudds R.L., Thomas A.L.R. (2003). Flying and swimming animals cruise at a Strouhal number tuned for high power efficiency. Nature.

[17] Lua K.B., Lim T.T., Yeo K.S., Oo G.Y. (2007). Wake-structure formation of a heaving two-dimensional elliptic airfoil. AIAA J..

[18] Anderson J., Streitlien K., Barrett D., Triantafyllou M. (1998). Oscillating foils of high propulsive efficiency. J. Fluid Mech..

[19] Bomphrey R.J., Nakata T., Henningsson P., Lin H.T. (2016). Flight of the dragonflies and damselflies. Philos. Trans. R. Soc. B-Biol. Sci..

[20] Liu X., Hefler C., Shyy W., Qiu H. (2021). The Importance of Flapping Kinematic Parameters in the Facilitation of the Different Flight Modes of Dragonflies. J. Bionic Eng..

[21] Lian Y., Broering T., Hord K., Prater R. (2014). The characterization of tandem and corrugated wings. Prog. Aeosp. Sci..

[22] Gong W.Q., Jia B.B., Xi G. (2016). Experimental study on instantaneous thrust and lift of two plunging wings in tandem. Exp. Fluids.

[23] Azuma A., Watanabe T. (1988). Flight performance of a dragonfly. J. Exp. Biol..

[24] Wang Z.J. (2004). The role of drag in insect hovering. J. Exp. Biol..

[25] Norberg R.A., Wu T.Y., Brokaw C.J., Brennen C. (1975). Hovering flight of the dragonfly *Aeschna juncea* L., kinematics and aerodynamics. Swimming and Flying in Nature.

[26] Alexander D. (1984). Unusual Phase-Relationships between the Forewings and Hindwings in Flying Dragonflies. J. Exp. Biol..

[27] Thomas A.L.R., Taylor G.K., Srygley R.B., Nudds R.L., Bomphrey R.J. (2004). Dragonfly flight: Free-flight and tethered flow visualizations reveal a diverse array of unsteady lift-generating mechanisms, controlled primarily via angle of attack. J. Exp. Biol..

[28] Lan S., Sun M. (2001). Aerodynamic force and flow structures of two airfoils in flapping motions. Acta Mech. Sin..

[29] Lua K.B., Lu H., Zhang X.H., Lim T.T., Yeo K.S. (2016). Aerodynamics of two-dimensional flapping wings in tandem configuration. Phys. Fluids.

[30] Zheng Y., Wu Y., Tang H. (2016). An experimental study on the forewing-hindwing interactions in hovering and forward flights. Int. J. Heat Fluid Flow.

[31] Muscutt L.E., Weymouth G.D., Ganapathisubramani B. (2017). Performance augmentation mechanism of in-line tandem flapping foils. J. Fluid Mech..

[32] Peng L., Zheng M., Pan T., Su G., Li Q. (2021). Tandem-wing interactions on aerodynamic performance inspired by dragonfly hovering. R. Soc. Open Sci..

[33] Gong W.Q., Jia B.B., Xi G. (2015). Experimental Study on Mean Thrust of Two Plunging Wings in Tandem. AIAA J..

[34] Nagai H., Fujita K., Murozono M. (2019). Experimental Study on Forewing-Hindwing Phasing in Hovering and Forward Flapping Flight. AIAA J..

[35] Boschitsch B.M., Dewey P.A., Smits A.J. (2014). Propulsive performance of unsteady tandem hydrofoils in an in-line configuration. Phys. Fluids.

[36] Heydari S., Kanso E. (2021). School cohesion, speed and efficiency are modulated by the swimmers flapping motion. J. Fluid Mech..

[37] Chen Z., Li X., Chen L. (2022). Enhanced performance of tandem plunging airfoils with an asymmetric pitching motion. Phys. Fluids.

[38] Lin X., Wu J., Zhang T., Yang L. (2021). Flow-mediated organization of two freely flapping swimmers. J. Fluid Mech..

[39] Joshi V., Mysa R.C. (2021). Mechanism of wake-induced flow dynamics in tandem flapping foils: Effect of the chord and gap ratios on propulsion. Phys. Fluids.

[40] Fry S.N., Sayaman R., Dickinson M.H. (2005). The aerodynamics of hovering flight in Drosophila. J. Exp. Biol..

[41] Willmott A.P., Ellington C.P. (1997). The mechanics of flight in the hawkmoth Manduca sexta I. Kinematics of hovering and forward flight. J. Exp. Biol..

[42] Wakeling J.M., Ellington C.P. (1997). Dragonfly flight: II. Velocities, accelerations and kinematics of flapping flight. J. Exp. Biol..

[43] Chen Y.H., Skote M., Zhao Y., Huang W.M. (2013). Dragonfly (*Sympetrum flaveolum*) flight: Kinematic measurement and modelling. J. Fluids Struct..

[44] Zhou C., Wu J. (2021). Kinematics, Deformation, and Aerodynamics of a Flexible Flapping Rotary Wing in Hovering Flight. J. Bionic Eng..

[45] Wang C., Zhou C., Zhu X. (2020). Influences of flapping modes and wing kinematics on aerodynamic performance of insect hovering flight. J. Mech. Sci. Technol..

[46] Amiralaei M.R., Alighanbari H., Hashemi S.M. (2011). Flow field characteristics study of a flapping airfoil using computational fluid dynamics. J. Fluids Struct..

[47] Esfahani J.A., Barati E., Karbasian H.R. (2015). Fluid structures of flapping airfoil with elliptical motion trajectory. Comput. Fluids.

[48] Yang S., Liu C., Wu J. (2017). Effect of motion trajectory on the aerodynamic performance of a flapping airfoil. J. Fluids Struct..

[49] Zhang R., Zhou C., Wang C., Xie P. (2017). Aerodynamic characteristics of dragonfly in asymmetric flapping. Acta Aeronaut. Astronaut. Sin..

[50] Li Q., Zheng M., Pan T., Su G. (2018). Experimental and Numerical Investigation on Dragonfly Wing and Body Motion during Voluntary Take-off. Sci. Rep..

[51] Wang C., Zhang R., Zhou C., Sun Z. (2020). Numerical Investigation on Flapping Aerodynamic Performance of Dragonfly Wings in Crosswind. Int. J. Aerosp. Eng..

[52] Lee Y.J., Lua K.B., Lim T.T., Yeo K.S. (2016). A quasi-steady aerodynamic model for flapping flight with improved adaptability. Bioinspir. Biomim..

[53] Broering T.M., Lian Y.S. (2012). The effect of phase angle and wing spacing on tandem flapping wings. Acta Mech. Sin..

[54] Tuncer I.H., Kaya M. (2003). Thrust generation caused by flapping airfoils in a biplane configuration. J. Aircraft.

[55] Miao J.M., Ho M.H. (2006). Effect of flexure on aerodynamic propulsive efficiency of flapping flexible airfoil. J. Fluids Struct..

